# Erythrocyte *PIG‐A* mutant frequencies in cancer patients receiving cisplatin

**DOI:** 10.1002/cam4.6895

**Published:** 2024-01-12

**Authors:** Vasily N. Dobrovolsky, Omar T. Atiq, Robert H. Heflich, Mackean Maisha, Page B. McKinzie, Mason G. Pearce, Timothy W. Robison

**Affiliations:** ^1^ Division of Genetic and Molecular Toxicology National Center for Toxicological Research (NCTR), US Food and Drug Administration (FDA) Jefferson Arkansas USA; ^2^ University of Arkansas for Medical Sciences (UAMS) Winthrop P. Rockefeller Cancer Institute Little Rock Arkansas USA; ^3^ Office of Scientific Coordination, NCTR, FDA Jefferson Arkansas USA; ^4^ Office of New Drugs, OII, DPTII, Center for Drug Evaluation and Research (CDER), US FDA Silver Spring Maryland USA

**Keywords:** chemotherapy, erythrocytes, head and neck cancer (HNC), mutations, Phosphatidylinositol glycan class‐A (*PIG‐A*) gene

## Abstract

**Background:**

Cisplatin is a primary chemotherapy choice for various solid tumors. DNA damage caused by cisplatin results in apoptosis of tumor cells. Cisplatin‐induced DNA damage, however, may also result in mutations in normal cells and the initiation of secondary malignancies. In the current study, we have used the erythrocyte *PIG‐A* assay to evaluate mutagenesis in non‐tumor hematopoietic tissue of cancer patients receiving cisplatin chemotherapy.

**Methods:**

Twenty‐one head and neck cancer patients undergoing treatment with cisplatin were monitored for the presence of *PIG‐A* mutant total erythrocytes and the young erythrocytes, reticulocytes (RETs), in peripheral blood for up to five and a half months from the initiation of the anti‐neoplastic chemotherapy.

**Results:**

*PIG‐A* mutant frequency (MF) in RETs increased at least two‐fold in 15 patients at some point of the monitoring, while the frequency of total mutant RBCs increased at least two‐fold in 6 patients. A general trend for an increase in the frequency of mutant RETs and total mutant RBCs was observed in 19 and 18 patients, respectively. Only in one patient did both RET and total RBC *PIG‐A* MFs did not increase at any time‐point over the monitoring period.

**Conclusion:**

Cisplatin chemotherapy induces moderate increases in the frequency of  *PIG‐A* mutant erythrocytes in head and neck cancer patients. Mutagenicity measured with the flow cytometric *PIG‐A* assay may serve as a tool for predicting adverse outcomes of genotoxic antineoplastic therapy.

## Abstract 2

Cisplatin chemotherapy induces moderate increases in the frequency of *PIG‐A* mutant erythrocytes in head and neck cancer patients. Mutagenicity measured with the flow cytometric *PIG‐A* assay may serve as a tool for predicting adverse outcomes of genotoxic antineoplastic therapy.

## 1 INTRODUCTION

The rodent erythrocyte Phosphatidylinositol glycan class‐A gene (*Pig‐a*) mutation detection assay is used by the US Food and Drug Administration as a test for monitoring in vivo mutagenicity in nonclinical safety studies.^1^, ^2^, ^3^ The rodent assay was developed on the shoulders of the human *PIG‐A* assay described by Araten et al.^4^, ^5^ in 1999, which itself was developed from an in‐depth understanding of the molecular basis of the human acquired genetic disease, paroxysmal nocturnal hemoglobinuria, or PNH, and the ex vivo properties of blood cells derived from PNH patients.^6^, ^7^, ^8^, ^9^, ^10^ The sensitivity and throughput of the original rodent red blood cell (RBC) assay were improved by using fluorescent staining for detecting *Pig‐a* mutant reticulocytes (RETs) and the development of methods for magnetic enrichment (ME) of mutants^11^, ^12^ or the RET fraction.^13^ As both the rodent and human assays are based on the same principle, similar sensitivity and throughput improvements were incorporated into the human assay.^14^

The performance of the rodent *Pig‐a* assay was validated in interlaboratory trials and in multiple nonclinical safety studies^15^, ^16^, ^17^, ^18^, ^19^; however, the performance of the advanced human *PIG‐A* assay for detecting mutagenicity induced by exogenous agents has not been confirmed. Several studies that have been conducted using human RBC *PIG‐A* assays were deficient in one way or another. It was demonstrated that workers operating in an environment where potentially hazardous chemicals (i.e., polycyclic aromatic hydrocarbons) are present have higher *PIG‐A* MFs than workers operating in a clean environment, e.g., in the office.^20^ Other studies observed that cancer patients treated with anti‐neoplastic chemotherapies on average have higher *PIG‐A* MFs than self‐identified healthy people.^21^, ^22^ But these studies did not measure *PIG‐A* MFs in the same subjects before being exposed to hazardous environments or anti‐cancer drugs, and causality between the exposure/treatment and elevated *PIG‐A* MFs was not demonstrated directly. In the studies where *PIG‐A* MFs were measured before and after exposure, the number of study subjects was insufficient for making conclusions.^23^

In the current study, we have verified the performance of the advanced human erythrocyte *PIG‐A* assay in a longitudinally controlled study. RET and total RBC *PIG‐A* MFs were evaluated in a large group of cancer patients before the start of chemotherapy, at several time points during chemotherapy, and after the completion of the chemotherapy regimen.

The study had several objectives: 1. To determine if the human erythrocyte *PIG‐A* assay is sufficiently sensitive for detecting mutagenicity caused by therapeutic doses of a known genotoxin, cisplatin, which has been shown to induce *Pig‐a* mutant erythrocytes in the rodent assay; 2. To determine if the success or failure of cisplatin anti‐cancer effect is related in any way to the change in erythrocyte *PIG‐A* MF during the chemotherapy regimen; and 3. To lay a foundation for a long‐term study associating the value of *PIG‐A* MF in response to the relatively short‐term treatment with cisplatin and the potential for tumor recurrence or induction of secondary tumors in the same cancer patients in the future.

## 2 MATERIALS AND METHODS

### 2.1 Study subjects

All procedures related to patient recruitment for the study, procedures for handling biological samples, and the patient informed consent form were reviewed and approved by the University of Arkansas for Medical Sciences (UAMS) Institutional Review Board and the FDA Research Involving Human Subjects Committee. Patient recruitment occurred at the UAMS Cancer Institute. The recruited patients were allowed to leave the study at any stage without any precondition. Study subjects were newly diagnosed cancer patients, for whom cisplatin chemotherapy was prescribed as a part of the complete therapy regimen. Patients receiving concomitant targeted radiotherapy were included in the study population as long as major sites of hematopoiesis were not exposed to ionizing radiation. Head and neck cancer (HNC) patients recommended for a standard 3 × 100 mg/m^2^ cisplatin infusion chemotherapy regimen, with or without radiation, were the preferred cohort. The study subjects agreed to provide a blood sample at each cisplatin infusion cycle and an additional sample at a follow‐up visit 1–5 months after the last infusion cycle (for a total of four blood samples). Peripheral blood was collected at the Cancer Institute; approximately 6 mL of peripheral blood was drawn from the saphenous vein into a standard EDTA‐K_2_ Vacutainer tube.

### 2.2 Erythrocyte *PIG‐A* analyses

Phosphatidylinositol glycan class‐A analyses were performed at the National Center for Toxicological Research (NCTR). In most cases, blood was processed within 2–3 h of collection. In cases when the blood sample was collected late in the day, the vacutainer tube with blood was stored overnight at +4°C and processed for the *PIG‐A* assay the next morning. Erythrocyte *PIG‐A* assays were performed using the In Vivo MutaFlow PLUS‐25H kit (Litron, Rochester, NY) following the manufacturer‐suggested protocol. Gradient centrifugation on Lympholyte®‐H cell separation medium (Cedarlane, Burlington, NC) was used for leucodepletion. Anti‐PE magnetic beads, LS magnetic columns, and quadroMACS™ magnet were used for magnetic enrichment of the mutant fraction (all from Miltenyi Biotec, Gaithersburg, MD). CountBright™ beads (Thermo Fisher Scientific; Waltham MA) were used in flow cytometric analyses for determining cell‐equivalents and calculating RET and total RBC MFs as suggested by Litron. Flow cytometry was performed on a FACSCanto™ II analyzer equipped with 488‐nm and 633‐nm lasers and operating under the control of FACSDiva™ v.9 data acquisition software (BD Biosciences; San Jose, CA). All *PIG‐A* assays were performed in triplicate to increase the accuracy of MF measurement. Each replicate utilized 200 μL of anticoagulant‐preserved blood (for a total of 600 μL of blood for the three replicates assayed at each time point). In a typical blood sample, each replicate generated mutant frequency data on 2–3 × 10^8^ total RBC equivalents and 3–8 × 10^6^ RET equivalents.

### 2.3 Statistical analyses

The average values of observed triplicate values of the RET MFs and total RBC MFs for each time point were used in analyses and log‐transformations. MFs were accessed for normality violations using the Kolmogorov–Smirnov, Cramer‐von Misses, and Anderson‐Darling tests. A one‐way analysis of variance (ANOVA) mixed model was used to evaluate the relationship between *PIG‐A* MF and observation time. Pairwise multiple comparisons between the observation time points were evaluated using the post hoc Dunn–Šidák correction to control the Type I error rate. The non‐parametric Jonckheere–Terpstra test was used to evaluate the overall trend in *PIG‐A* MFs over increasing time post‐exposure to cisplatin. All statistical tests were two‐sided, unless otherwise stated, and significance was assessed at the nominal 0.05 level. SigmaPlot 14.5 (Inpixon; Palo Alto, CA) and SAS for Windows v9.3 (SAS; Cary, NC) software were used for statistical analyses.

To perform pairwise comparisons, *PIG‐A* assay results were grouped in four categories based on the patients' office visit for blood donation and/or cisplatin infusion. Group 1 (pre‐treatment; *N* = 21) were blood samples from patients visiting the oncology clinic for the first cisplatin infusion and the first blood collection (Day 0 of the study; cisplatin infusion was administered after blood collection). Group 2 (*N* = 21) were samples from the same patients during their visit for the second cisplatin infusion (approximately Day 21 of the study; the second blood sample was collected before cisplatin was administered). Group 3 (*N* = 20) samples were from the third office visit of patients that remained in the study (approximately Day 42 of the study; cisplatin infusion was administered on this day but not to all patients). Group 4 (*N* = 15) were from the fourth visit of patients that remained on the study (Days 70–159 of the study; no infusion was administered on this visit). Grouping was proportional but not necessarily linear with regards to cumulative dose of cisplatin received (see Table 1 for details of cisplatin treatment).

**TABLE 1:** Patient demographics, diagnostics, and treatment with cisplatin chemotherapy.

Patient ID	Sex	Age	Cancer staging	Primary tumor site	Prescribed chemotherapy regimen	Response to cisplatin	Smoker	Other treatment	Comments
HL001	Male	41	IVB	Tongue	Standard^a^	UNK^b^	Yes		Went off study after the third dose of cisplatin
RJ002	Female	60	III	Floor of mouth	Standard	NOD^c^	Yes (former)	Radiation	
WM003	Male	46	II	Right tonsil	Standard (final dose not administered)	NOD	No	Radiation	Final dose not administered due to nausea and bilateral tinnitus
SB004	Male	51	III	Oropharynx/ base of tongue	Standard (final dose not administered)	PRT^d^	No	Radiation	Final dose not administered due to infected infusaport
CH005	Female	59	III	Larynx	Standard	NOD	Yes	Radiation	
KD009	Female	32	III	Cervix	Modified; see comments	NOD	No	Radiation	Adjusted cisplatin schedule: 70 mg on Days 0, 7, 14, 21, 28, and 37
AW010	Male	39	III	Larynx	Standard (final dose not administered)	NOD	Yes	Radiation	Final dose not administered due to severe bilateral tinnitus
MM011	Male	50	IV	Oral cavity	Standard (final dose not administered)	UNK	Yes	Radiation	Went off the study after the second dose, passed away 9 months later
DW012	Male	48	IV	Tongue	Standard	NOD	Yes	Radiation	
GM013	Male	63	IV	Tongue	Standard	STD^e^	Yes	Radiation	
WM014	Male	56	IVB	Larynx	Standard	NOD	Yes		
JR015	Male	49	IVA	Mandible	Standard	NOD	No	Radiation	
CC016	Male	61	I	Oropharynx	Standard	NOD	Yes	Radiation	
LH017	Female	74	II	Oropharynx	Modified; see comments	NOD	Yes	Radiation	Adjusted cisplatin schedule: 100 mg/m^2^ for cycle 1; 50 mg/m^2^ for cycles 2 and 3; 100 mg/m^2^ for cycle 4
RR018	Male	86	III	Oropharynx	Standard (final dose not administered)	NOD	No	Radiation	Final dose not administered, passed away 2 months after the second dose
CM019	Female	42	II	Base of tongue	Standard (final dose not administered)	UNK	Yes	Radiation	Refused the final dose, went off study
NB020	Male	56	IVA	Tongue	Standard	NOD	Yes	Radiation	
AR021	Male	56	III	Tonsil	Standard	NOD	No	Radiation	
MW022	Female	70	IVA	Hypopharynx	Standard (final dose not administered)	NOD	No	Radiation	
SR023	Male	48	III	Oropharynx	Standard	PRT	No	Radiation	
WM024	Male	56	II	Base of tongue	Standard	NOD	Yes	Radiation	

*Note*: In all patients the type of cancer was identified as squamous cell carcinoma. Ethnic origin is believed to be irrelevant for this study; this information is not shown in the Table. Patients SL006, SR007, and TJ008 withdrew from the study after the first cycle of cisplatin therapy.

^a^ Standard prescribed chemotherapy regimen: 3 cycles of 100 mg/m^2^ cisplatin infusion every 3 weeks (i.e., on Days 0, 21, and 42; with Day 0 being the day of the first infusion). The actual visit days for the second and the third infusions of cisplatin varied by up to 6 days (see Table 2). Patients KD009 and LH017 had modified schedules and doses of cisplatin therapy.

^b^ UNK: unknown.

^c^ NOD: no evidence of disease at the end of study.

^d^ PRT: partial response.

^e^ STD: stable disease.

## 3 RESULTS

**FIGURE 1:**
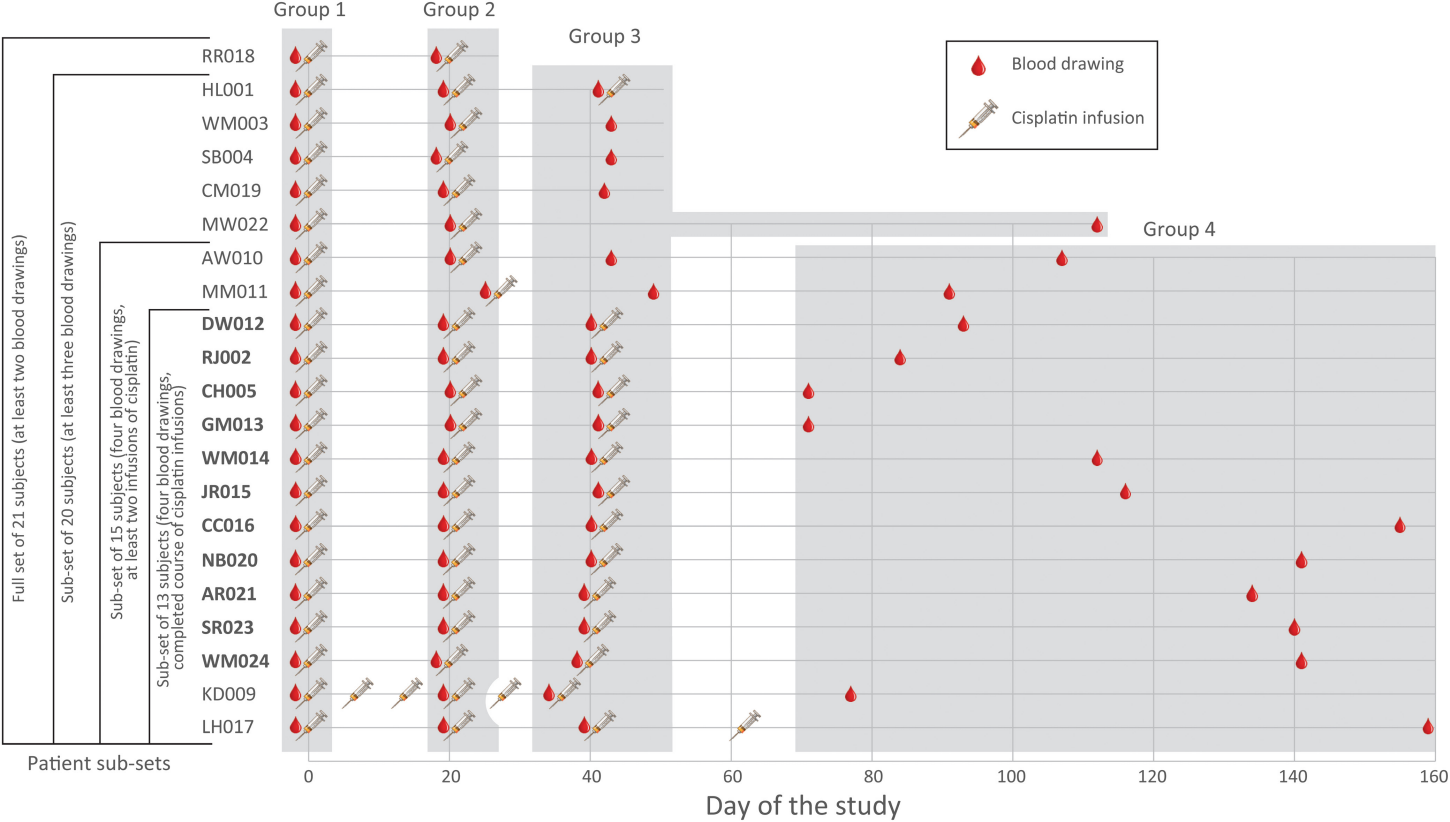
Blood drawing and cisplatin infusion timing relative to day of the study. Not all subjects submitted the requested four blood samples; not all subjects completed cisplatin chemotherapy course. The therapy course was not the same for all subjects. The vertical order of patients is based on the number of donated blood samples and the number of cisplatin infusions. Groups were based on office visits for blood sampling. The 11 patients highlighted in bold received the standard 3‐infusion cisplatin therapy course and submitted the requested four blood samples. For details, see Results and Table 1.

Twenty‐four cancer patients were recruited for the erythrocyte *PIG‐A* study (see Table 1 for complete description). Twenty‐three patients were diagnosed with various stages of HNC, and one female was diagnosed with cervical cancer. All cases (including the cervical cancer) were typed as squamous cell carcinoma. The patients were from different ethnic backgrounds (16 white, 6 black, and 2 other) and ranged from 32 to 86 years of age. Eighteen patients were males and six were females; 16 patients were identified as former or current smokers. For all HNC patients, a standard chemotherapy regimen consisted of 3 cycles of 100 mg/m^2^ cisplatin infusion (one infusion every 3 weeks). The patient with cervix squamous cell carcinoma received a different chemotherapy regimen, with more frequent infusions of lower cisplatin doses. Three patients withdrew from the study after the first cycle of cisplatin infusion, submitting only a Day 0 blood sample; these three patients were not included in the analyses. Not all HNC patients received the complete prescribed regimen of cisplatin for various reasons (some withdrew from the study, while others refused the final dosing). Only 15 patients submitted the requested four blood samples (including the sample at a follow‐up visit). Only 11 patients received all three prescribed cycles of cisplatin infusion (at regular intervals) and provided the requested 4 blood samples. The schedule of blood collections relative to cisplatin infusions for all patients is shown in Figure 1. Of the 21 patients remaining on the study after the first cycle of cisplatin infusion, 15 did not show evidence of disease at the end of the study; some of these did not complete the full 3‐cycle chemotherapy regimen. In three patients, the response to cisplatin therapy was either partial or negative; in another three patients, the outcome of chemotherapy could not be determined because they withdrew from the study after the second or third infusion cycle and didn't return for the follow‐up visit.

**FIGURE 2:**
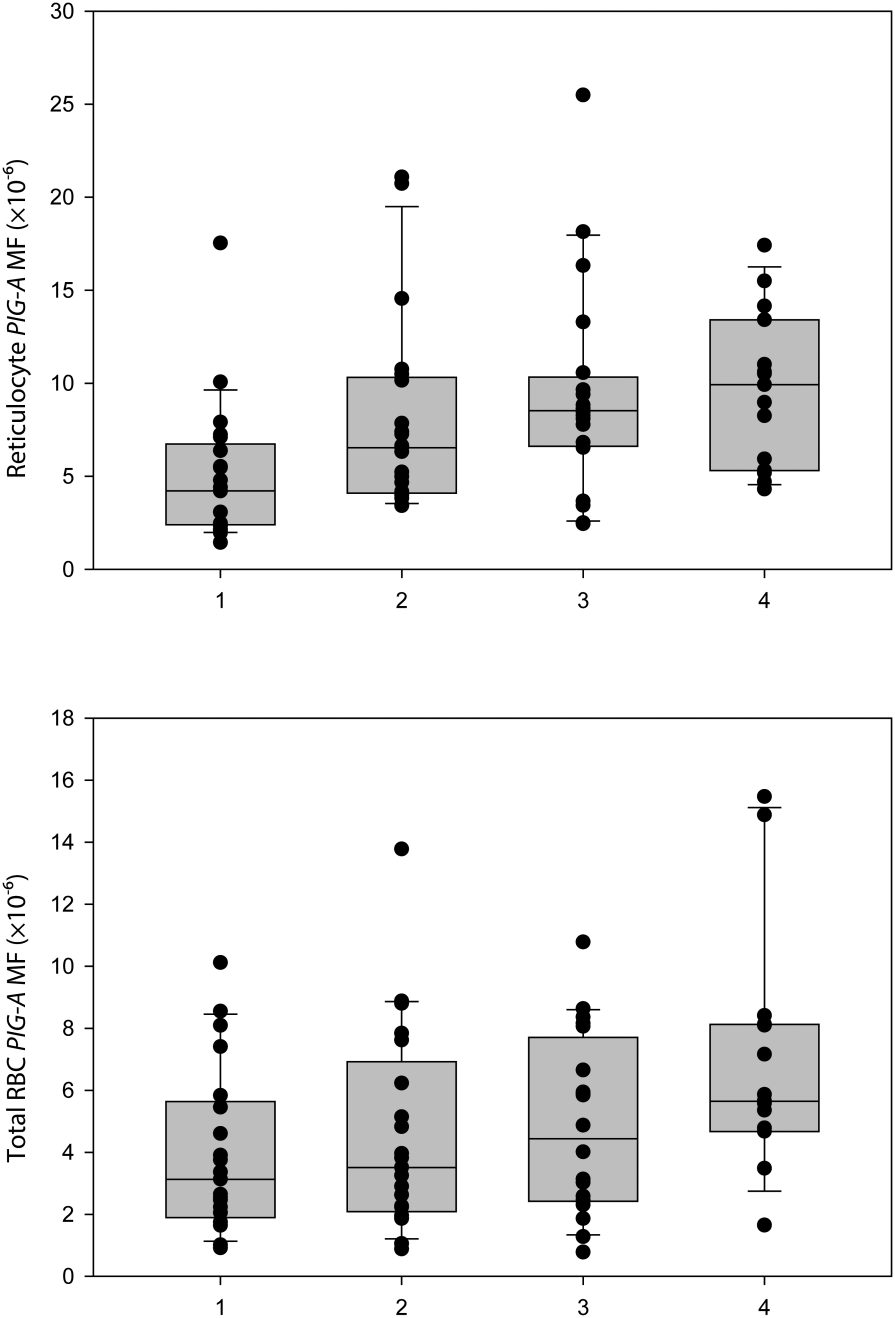
Box and whiskers percentile charts for reticulocyte (RET) and total RBC absolute *PIG‐A* mutant frequencies (MF) measured in cancer patients receiving cisplatin. The boundaries of the box represent the 25th and 75th percentiles; the horizontal line within the box marks the median; and the whiskers indicate the 10th and 90th percentiles. MFs were determined at scheduled office visits for cisplatin infusions or for follow‐up post‐therapy visits at which blood samples were collected (Groups 1, 2, 3, and 4; see Table 1 and Materials & Methods for details on timing of office visits). MFs are shown for all 21 patients (fewer blood samples were collected at the third and the fourth office visits as some patients withdrew from the study; see Table 1). Data for the charts are taken from Table 2.

**FIGURE 3:**
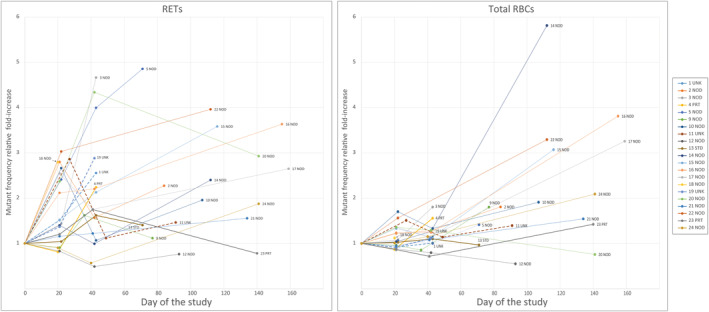
Time‐dependent relative fold‐increases in RET and total RBC *PIG‐A* MFs in cancer patients receiving cisplatin chemotherapy. Each data point was calculated as the ratio of the *PIG‐A* MF determined at a specific day of study and the MF determined at Day 0 (the first office visit) before cisplatin infusion (data for the charts are taken from Table 2). Only the numerical index of the patient ID and the disease status at the end of the study are shown in the charts. Data points for each patient are traced by lines. Dotted lines represent patients with unknown status of disease at the end of the study.

Reticulocyte and total RBC *PIG‐A* MFs determined at time points the patients consented for submitting blood samples are shown in Table 2. The raw RET and total RBC *PIG‐A* MFs for patients grouped by office visit are shown in Figure 2. Also, fold‐change values relative to the pre‐treatment baseline values were computed for exploratory visual assessments (see Figure 3). No statistical tests were performed on this measure; however, a fold‐change value of about 2 was considered of interest. In most patients, *PIG‐A* MF was increased in both fractions of RBCs at one or more time points relative to the pre‐treatment time point, Day 0. A somewhat earlier response in the induction of *PIG‐A* MFs was observed for the RET fraction.

**TABLE 2:** Frequencies of *PIG‐A* mutant reticulocytes (RETs) and mutant total red blood cells (RBCs) in peripheral blood of cancer patients receiving cisplatin chemotherapy.

Patient ID and response to cisplatin^b^	Day of blood draw	RET MF^a^ (×10^−6^)		Total RBC MF^a^ (×10^−6^)	
		Measured in replicates	Mean	Measured in replicates	Mean
HL001 UNK	0	8.25; 5.86; 7.18	7.10	9.54; 7.62; 8.48	8.55
	21	13.36; 8.59; 10.29	10.75	8.81; 7.26; 7.45	7.84
	43	20.90; 17.74; 15.79	18.14	8.64; 8.30; 8.95	8.63
RJ002 NOD	0	3.40; 2.44; 1.16	2.33	3.44; 3.03; 2.90	3.13
	21	6.35; 7.52; 5.76	6.54	4.36; 3.26; 3.85	3.82
	42	2.61; 3.79; 4.57	3.66	3.79; 3.69; 4.55	4.01
	84	7.06; 4.63; 4.24	5.31	6.01; 5.30; 5.63	5.65
WM003 NOD	0	7.85; 4.24; 4.30	5.46	1.97; 1.49; 1.75	1.74
	22	8.00; 6.54; 9.00	7.85	1.71; 1.75; 2.17	1.88
	43^c^	30.37; 19.89; 26.20	25.49	3.38; 2.94; 3.08	3.13
SB004 PRT	0	4.28; 3.84; 4.48	4.20	3.76; 4.08; 3.40	3.75
	20	4.43; 2.04; 3.78	3.42	3.57; 3.20; 2.99	3.25
	43^c^	9.56; 9.43; 9.23	9.40	8.25; 4.29; 4.97	5.84
CH005 NOD	0	1.67; 3.05; 1.77	2.16	2.36; 2.54; 2.48	2.46
	22	6.57; 3.61; 5.48	5.22	2.78; 2.24; 2.83	2.62
	43	10.59; 7.58; 7.77	8.64	2.58; 2.39; 2.42	2.46
	71	11.32; 8.31; 11.89	10.51	3.67; 3.30; 3.47	3.48
KD009 NOD	0	3.76; 4.36; 4.50	4.21	0.77; 0.97; 1.01	0.92
	21	3.76; 2.61; 5.08	3.82	0.87; 0.85; 0.92	0.88
	36	5.24; 9.71; 5.51	6.82	0.70; 0.91; 0.74	0.78
	77	4.12; 4.01; 5.96	4.70	1.66; 1.55; 1.73	1.65
AW010 NOD	0	8.33; 7.35; 8.06	7.91	8.95; 7.88; 7.45	8.09
	22	21.73; 23.05; 18.44	21.08	13.45; 16.50; 11.40	13.78
	43^c^	8.32; 6.70; 10.33	8.45	10.65; 11.38; 10.29	10.78
	107	13.83; 15.50; 17.15	15.49	14.82; 15.24; 16.35	15.47
MM011 UNK	0	6.78; 8.28; 6.66	7.24	6.26; 5.51; 5.72	5.83
	27	26.97; 15.82; 19.40	20.73	10.11; 8.43; 7.87	8.80
	49^c^	7.93; 8.71; 7.65	8.09	6.56; 6.93; 6.46	6.65
	91	10.51; 11.27; 10.03	10.61	8.60; 7.90; 7.89	8.13
DW012 NOD	0	14.28; 20.15; 18.15	17.53	7.97; 12.26; 10.12	10.12
	21	13.59; 12.39; 17.66	14.55	8.93; 8.64; 9.06	8.88
	42	4.25; 8.95; 12.60	8.60	7.77; 7.91; 8.50	8.06
	93	15.41; 13.43; 11.38	13.41	5.93; 5.41; 5.40	5.58
GM013 STD	0	6.28; 13.06; 10.87	10.07	7.44; 6.69; 8.11	7.41
	22	10.69; 8.83; 11.96	10.49	7.46; 7.05; 8.34	7.62
	43	18.03; 16.75; 14.17	16.32	8.31; 8.02; 8.15	8.16
	71	13.67; 13.60; 15.18	14.15	6.86; 6.66; 7.96	7.16
WM014 NOD	0	2.08; 2.25; 3.08	2.47	0.99; 0.96; 1.08	1.01
	21	4.30; 2.26; 3.82	3.46	1.05; 0.84; 1.25	1.05
	42	1.16; 1.72; 4.48	2.45	1.26; 1.17; 1.40	1.28
	112	6.69; 5.89; 5.22	5.93	5.46; 6.54; 5.60	5.86
JR015 NOD	0	3.18; 3.11; 2.92	3.07	2.46; 2.30; 3.14	2.64
	21	3.15; 4.32; 6.55	4.67	3.50; 3.09; 3.96	3.51
	43	6.23; 5.62; 7.77	6.54	3.03; 2.97; 3.05	3.02
	116	14.82; 9.55; 8.66	11.01	9.23; 8.30; 6.76	8.10
CC016 NOD	0	3.82; 4.13; 6.42	4.79	3.61; 3.60; 4.49	3.90
	21	8.33; 11.26; 10.86	10.15	4.09; 5.33; 5.03	4.82
	42	13.72; 9.94; 8.03	10.56	5.55; 5.08; 3.98	4.87
	155	17.24; 16.54; 18.46	17.41	13.49; 15.20; 15.96	14.88
LH017 NOD	0	2.16; 1.65; 2.06	1.96	1.46; 1.42; 2.05	1.64
	21	5.41; 3.66; 5.87	4.98	2.62; 2.13; 1.96	2.23
	41	4.06; 1.97; 4.26	3.43	2.01; 1.68; 1.90	1.86
	159	5.84; 5.51; 4.19	5.18	4.43; 5.75; 5.88	5.35
RR018 NOD	0	1.35; 1.00; 1.96	1.44	1.50; 1.71; 2.02	1.74
	20	5.22; 2.58; 4.23	4.01	1.87; 2.11; 1.88	1.95
CM019 UNK	0	2.55; 2.53; 4.10	3.06	2.08; 2.15; 1.92	2.05
	21	5.09; 3.44; 4.03	4.18	1.87; 1.88; 1.84	1.86
	42^c^	8.39; 9.24	8.82	2.10; 2.49	2.30
NB020 NOD	0	2.65; 4.18; 2.36	3.06	4.55; 4.53; 4.72	4.60
	21	6.08; 6.47; 9.24	7.26	5.66; 6.30; 6.75	6.23
	42	13.79; 10.90; 15.19	13.29	6.53; 5.30; 6.00	5.94
	141	10.60; 7.69; 8.62	8.97	3.28; 3.95; 3.21	3.48
AR021 NOD	0	6.48; 7.00; 5.64	6.37	5.78; 5.18; 5.38	5.45
	21	7.19; 6.56; 8.41	7.39	5.02; 4.99; 5.42	5.14
	41	10.01; 6.48; 6.84	7.78	6.07; 5.46; 6.13	5.89
	134	10.97; 10.96; 7.84	9.92	8.27; 9.12; 7.84	8.41
MW022 NOD	0	1.14; 1.85; 3.26	2.08	2.39; 2.85; 2.36	2.54
	22	7.13; 5.43; 6.39	6.32	5.05; 3.52; 3.31	3.96
	112	8.84; 8.23; 7.71	8.26	9.66; 8.36; 7.04	8.36
SR023 PRT	0	6.11; 5.44; 5.00	5.52	3.21; 3.50; 3.37	3.36
	21	6.11; 9.12; 4.68	6.64	2.87; 3.17; 2.67	2.90
	41	6.63; 9.94; 12.38	9.65	2.04; 2.66; 2.52	2.41
	140	4.23; 3.85; 4.86	4.31	4.95; 4.40; 5.00	4.78
WM024 NOD	0	3.29; 5.27; 4.64	4.40	2.17; 2.33; 2.20	2.23
	20	3.09; 4.61; 4.23	3.98	2.07; 2.22; 2.49	2.26
	40	2.22; 2.41; 2.87	2.50	2.38; 2.72; 2.63	2.58
	141	7.02; 8.45; 9.29	8.25	4.80; 4.47; 4.74	4.67

*Note*: The total of 79 *PIG‐A* assays were performed in triplicates; only one replicate failed (CM019, Day 42). The first three blood collections occurred on days of cisplatin infusions (i.e., on Days 0, ca. Day 21, and ca. Day 42), unless the third infusion was canceled for whatever reason. When blood collection day coincided with cisplatin infusion day, blood was drawn before the infusion. Blood collections post‐Day 43 occurred at follow up visits; no infusions were performed on these days. Not all patients provided the expected four blood sample and not all patients received the prescribed three infusions of cisplatin. KD009 had a different schedule of cisplatin infusion, and the third blood sample was collected on Day 36 instead of ca. Day 42. LH017 had 4 cycles of cisplatin infusion; the blood was not collected at the fourth infusion cycle. Maximum increase in RET MF relative to MF determined on Day 0 was recorded for WM003 on Day 43 of the study (~20 × 10^−6^); maximum increase in total RBC MF was recorded for CC016 on Day 155 (~11 × 10^−6^). Maximum fold‐increases in MFs relative to Day 0 were recorded for different patients (see Figure 3).

^a^ Mutant frequency, MF.

^b^ Response to cisplatin chemotherapy is clarified in Table 1.

^c^ No cisplatin infusion happened during the third visit; see explanation in Table 1.

Descriptive summary statistics are shown in Table 3. Raw MF data were not supportive of bell‐shaped normal distribution, as all applied normally tests were significant (*p* < 0.05). Hence, the MF data were log‐transformed to conform with the normal distribution. The mixed model ANOVA was the preferred method of analysis because of its robustness to slight deviations from normality and its flexibility to account for missing values under the assumption of “ignorable missingness”, i.e., missing at random or missing completely at random.^24^ Generally, there were positive linear trends in RET *PIG‐A* MF (*p* < 0.001) and in total RBC *PIG‐A* MF (*p* = 0.007) over time following cisplatin infusions (Table 3). The greatest increases in MFs for the RET fraction were noted at visit 3 (at Day 42 or more) and visit 4 (at Day 70 or more), and at visit 4 for the total RBC fraction.

**TABLE 3:** Descriptive summary statistics and trend analysis for RET and total RBC PIG‐A MFs.

Day, (Group)	*N*	RET *PIG‐A* MFs			Total RBC *PIG‐A* MFs		
		Mean	SEM^a^	MFC^b^	Mean	SEM^a^	MFC^b^
0, (1)	21	5.07	0.80	1.00	3.96	0.58	1.00
21, (2)	21	7.98	1.13	1.80	4.54	0.71	1.14
42, (3)	20	9.35	1.24	2.14	4.85	0.64	1.27
70, (4)	15	9.68	1.07	2.22	6.84	1.00	2.11
Jonckheere–Terpstra test for overall trend		*Z* ^c^ = 3.997, *N* ^d^ = 77, *p*‐value^e^ < 0.001			*Z* ^c^ = 2.452, *N* ^d^ = 77, *p*‐value^e^ = 0.007		

^a^ Standard error of mean.

^b^ Mean of fold change.

^c^ Value for *Z*‐statistic.

^d^ Sample size for the estimated *Z*‐statistic.

^e^ One‐sided *p*‐value.

The overall effect of cisplatin infusion exposure over time was significant for RET *PIG‐A* MF (*p* < 0.001) and total RBC *PIG‐A* MF (*p* < 0.0037) as determined by ANOVA mixed model analysis. The results of pairwise multiple comparisons between mean mutant frequencies at each time‐point following cisplatin infusions are summarized in Table 4. The RET MFs were significantly elevated in cancer patients at three or more weeks after the initial infusion of cisplatin (comparisons of Day 0 vs. Day 21, *p* = 0.0006; vs. Day 42, *p* = 0.0021; and vs. Day 70, *p* < 0.0001). The effect of cisplatin treatment on the total RBC *PIG‐A* MFs was not evident after the first and the second infusions (comparisons of Day 0 vs. Day 21, *p* = 0.1550; and vs. Day 42, *p* = 0.1041). Total RBC *PIG‐A* MFs became significantly increased relative to prior time‐points only at follow‐up visits at Day 70 or later of the study (comparisons of Day 70 vs. Day 0, *p* = 0.0032; vs. Day 21, *p* = 0.0106; and vs. Day 42, *p* = 0.0081). These observations are consistent with the presumption that in the human erythrocyte *PIG‐A* assay, RETs are the leading fraction for detecting mutagenicity and total RBCs are the lagging fraction, as is the case for the rodent *Pig‐a* assay.

**TABLE 4:** Statistical comparisons of RET and total RBC *PIG‐A* MFs in patients receiving cisplatin chemotherapy.

Orthogonal contrasts	RET *PIG‐A* MF			Total RBC *PIG‐A* MF		
	*F*_[3,20]_ ^a^ = 13.46; *p*‐value^b^ < 0.0001			*F*_[3,20]_ ^a^ = 6.21; *p*‐value^b^ = 0.0037		
	*t*‐value^c^	DF^d^	Adjusted. *p* ^e^	*t*‐value^c^	DF^d^	Adjusted *p* ^e^
Day 21 versus Day 0 (Group 2 vs. Group 1)	4.85	20	0.0006	2.37	20	0.1550
Day 42 versus Day 0 (Group 3 vs. Group 1)	4.30	20	0.0021	2.57	20	0.1041
Day 70 versus Day 0 (Group 4 vs. Group 1)	5.80	20	<0.0001	4.12	20	0.0032
Day 42 versus Day 21 (Group 3 vs. Group 2)	1.00	20	0.9100	1.10	20	0.8654
Day 70 versus Day 21 (Group 4 vs. Group 2)	2.28	20	0.1867	3.60	20	0.0106
Day 70 versus Day 42 (Group 4 vs. Group 3)	0.87	20	0.9515	3.72	20	0.0081

^a^ Estimated value of the *F*‐test statistic [numerator DF = 3; denominator DF = 20].

^b^ *p*‐value for one‐way ANOVA mixed model analysis.

^c^ Estimated value of the *t*‐test statistic.

^d^ Degrees of freedom.

^e^ *p*‐Values adjusted for multiple comparisons using the Dunn–Šidák correction.

## 4 DISCUSSION

Cisplatin, cis‐diammine dichloroplatinum (II) or cis‐PtCl_2_(NH_3_)_2_, is a primary chemotherapy choice for various solid tumors, including HNC, one of the top 10 types of cancer worldwide. The most common cisplatin‐containing treatment regimen for HNC, as recommended by the Radiation Therapy Oncology Group, is 100 mg/m^2^ on Days 1, 22, and 43, in combination with radiotherapy (reviewed in Brown et al.^25^). In many cases (and as seen in the current study), initial cisplatin chemotherapy produces immediate positive results, i.e., no evidence of disease at the completion of the therapy course.

In an aqueous environment, chlorine molecules can be displaced from cisplatin by water to produce positively charged mono‐ or di‐substituted complexes that serve as electrophiles for attack of electron‐rich *N7* purine positions of genomic DNA. The most frequent cisplatin‐induced DNA lesions are guanine adducts (at *N7*) and 1,2‐intrastrand GG crosslinks. Less frequent lesions are 1,3‐intrastrand crosslinks at GNG sequences and GC interstrand crosslinks that result in severe distortion of the DNA structure. The main mechanism for the antitumor activity of cisplatin involves the formation of adducts and crosslinks in genomic DNA, which activates endogenous DNA repair processes and, if DNA damage is too extensive for repair, leads to apoptosis, (reviewed in Galluzzi et al.^26^). A possible side effect of chemotherapy (besides incomplete apoptosis in tumor tissue) is induction of mutations in non‐target, i.e., non‐tumor, tissue. Cisplatin is classified by the International Agency for Research on Cancer as a Group 2A carcinogen, i.e., a probable human carcinogen. The induction of mutations in non‐tumor tissue of cancer patients treated with cisplatin may be a biomarker for the future recurrence of cancer or the emergence of secondary malignancies. An increase in MF may also be a biomarker for the responsiveness or resistance of secondary or recurrent tumors to future cisplatin chemotherapy.

The erythrocyte *PIG‐A* assay is one of the few practical assays for assessing mutagenesis in human cancer patients. Very little blood is required for performing the assay. As cisplatin is administered to patients as an intravenous infusion, it is certain that cisplatin reaches the bone marrow, where it can induce mutations in nucleated dividing erythroid precursors cells. Previous studies indicate that cisplatin induces mutant cells in the rat erythrocyte *Pig‐a* assay,^27^, ^28^, ^29^ thus, it is likely that cisplatin is also a human *PIG‐A* gene mutagen. However, the optimal timing for measuring erythrocyte *PIG‐A* MFs after exposure to a potential mutagen is not known, making it necessary to measure *PIG‐A* MFs at several time points. In the current study, these time points ranged from as early as possible after the first infusion of cisplatin to after completing the chemotherapy regimen.

Our human *PIG‐A* study did not contain a separate vehicle‐control group of subjects. For each patient, the sample processed at Day 0 before the first cisplatin infusion served as control for that patient. From the literature, it is known that background *PIG‐A* MFs in individual healthy human subjects remain within a narrow range when measured repeatedly over a period of months; however, the background MFs measured in different human subjects vary widely. Self‐identified healthy donors have total RBC *PIG‐A* MFs from 1 × 10^−6^ to 10 × 10^−6^, with occasional outliers having MFs from 100 × 10^−6^ to 300 × 10^−6^.^12^, ^23^ Therefore, grouping humans into control and treated groups could result in poor resolution between background *PIG‐A* MFs and cisplatin‐induced *PIG‐A* MFs. In addition, cancer patients in the current study varied by age, and finding age‐matched control subjects would be burdensome for the study.

Another complication for the study was that not all patients were able to provide the complete set of four blood samples that were requested at the start of the study. Thus, the original study protocol of 3 standard infusions of 100 mg/m^2^ cisplatin combined with 4 blood collections at predetermined time points (0, 3 weeks, 6 weeks, and 10 to 14 weeks) was not followed for many of the study subjects. There are various approaches for the statistical analyses of such incomplete datasets (e.g., paired t‐test or repeated measures ANOVA), but they would require performing analyses on reduced sub‐sets of patients, which would inevitably decrease the power of analyses. Hence, the ANOVA mixed model was employed to use the full *PIG‐A* MF datasets that were acquired.

Despite all the caveats and logistical difficulties in performing the study, we were able to detect a general trend for increasing *PIG‐A* MFs in cisplatin‐treated patients (see Table 3). However, the response to treatment in individual patients was relatively small: the maximum relative increase in MF in the RET fraction was under 6‐fold, and the maximum absolute increase was approximately 20 × 10^−6^. The maximum increase in MF for the total RBC fraction was less than 5‐fold, and the maximum absolute increase was just under 11 × 10^−6^. In one patient, DW012, neither RET nor total RBC *PIG‐A* MF increased over the study period. Similar modest increases in *PIG‐A* MF were observed in a small group of testicular cancer patients receiving cisplatin‐containing chemotherapy.^30^ However, the chemotherapy regimen in the testicular cancer study was different than in our HNC study.

The response in the *PIG‐A* assay to cisplatin treatment in cancer patients may reflect specific properties of the human erythrocyte *PIG‐A* assay. The *PIG‐A* assay was developed as an extension of a diagnostic procedure for characterizing PNH. In PNH, a mutation in the endogenous X‐linked *PIG‐A* gene in hematopoietic bone marrow stem cells causes a disruption in the synthesis of glycosyl phosphatidyl inositol (GPI), which makes erythrocytes originating from the mutant bone marrow stem cells deficient in CD55 and CD59 GPI‐anchored surface proteins vulnerable for complement‐mediated lysis.^31^ As the CD59/CD55‐deficient phenotype is measured in the human erythrocyte *PIG‐A* assay, it might be hypothesized that cisplatin‐induced GPI‐deficient *PIG‐A* mutant erythrocytes in cancer patients are under pressure for elimination by the complement system, as occurs in PNH patients.

The dynamics of *PIG‐A* mutant erythrocytes accumulation (due to mutagenicity with cisplatin or any other xenobiotic agent) and elimination of *PIG‐A* erythrocytes (due to immune surveillance) are not known for humans. In the case of PNH, evidence indicates that even the youngest cohort of *PIG‐A* mutant erythrocytes, RETs, is under pressure for complement‐mediated elimination.^32^ However, several lines of evidence suggest that such elimination of mutants is neither rapid nor 100% efficient: PNH patients have high levels of CD55/CD59‐deficient RBCs in circulation.^33^ Also, several cases have been documented of relatively high frequencies of *PIG‐A* mutant RBCs in self‐identified healthy donors, as well as in some cancer patients. In the current study, we used a version of the *PIG‐A* assay that detects mutants among total RBCs and RETs. The assumption was that *PIG‐A* mutant RETs are exposed to the complement system for a shorter period of time than are mature RBCs, so that RETs may be more sensitive than total RBCs for detecting the mutagenic effects of cisplatin chemotherapy. Our observations, using an advanced *PIG‐A* assay employing magnetic enrichment of mutant erythrocytes, suggest that the mutagenic effects of cisplatin chemotherapy in human patients can be detected in RETs as early as 3 weeks after the first exposure, while in total RBCs, cisplatin‐induced increases in MF were detected only at 10 weeks or longer after the first exposure.

Of interest is that, in the rat cisplatin study, *Pig‐a* MFs increased approximately 5‐fold in high‐dose treated animals, and the absolute increases in MF (approximately 20 × 10^−6^) were similar for all animals in the treated group.^27^ Perhaps the more robust and uniform *Pig‐a* response observed in the rat model of cisplatin‐induced mutagenesis was at least partially due to lack of complement‐mediated hemolysis of *Pig‐a* mutant RBCs. The maximum cumulative dose of cisplatin in the rat study (6 mg/kg) was comparable to the total dose used in this human study (3 × 100 mg/m^2^), but the rats did not have tumors, and a more acute regimen of cisplatin exposure was used. Yet, a non‐linear increase in RET MF was observed for the rats treated with a low dose of cisplatin,^27^ just as was observed for several subjects in our study (e.g., RJ002, AW010, MM011).

In conclusion, we observed moderate increases in erythrocyte *PIG‐A* MF in patients receiving cisplatin chemotherapy. This confirms that the human and rodent *Pig‐a*/*Pig‐a* assays measure the same relevant endpoint (mutation), while the rodent assay may be a bit more sensitive due to better control over the experimental conditions and timing for blood analyses. In our study, there was no clear relationship between cisplatin‐induced *PIG‐A* MFs and the short‐term outcome of chemotherapy; most patients exhibited no evidence of disease at the end of the study. Long‐term monitoring of the study subjects, including measuring *PIG‐A* MFs, may shed a light on the role of mutagenesis and DNA repair in tumor recurrence, the induction of secondary tumors, and resistance to cisplatin chemotherapy.

## AUTHOR CONTRIBUTIONS

**Vasily N. Dobrovolsky:** Conceptualization (lead); investigation (lead); methodology (lead); writing – original draft (lead). **Omar T. Atiq:** Resources (lead). **Robert H. Heflich:** Conceptualization (supporting); writing – review and editing (lead). **Mackean Maisha:** Data curation (supporting); formal analysis (supporting). **Page B. McKinzie:** Data curation (equal); formal analysis (equal). **Mason G. Pearce:** Investigation (supporting); methodology (supporting). **Timothy W. Robison:** Conceptualization (equal).

## CONFLICT OF INTEREST STATEMENT

The authors declare no conflict of interests.

## DISCLAIMER

This manuscript reflects the views of the authors and does not necessarily reflect those of the U.S. Food and Drug Administration. Any mention of commercial products is for clarification only and is not intended as approval, endorsement, or recommendation.

## Data Availability

n/aAll data described and presented in the manuscript is original, it will be available world‐wide upon acceptance of the manuscript.
